# Unit with Fluidized Bed for Gas-Vapor Activation of Different Carbonaceous Materials for Various Purposes: Design, Computation, Implementation

**DOI:** 10.1186/s11671-017-1843-0

**Published:** 2017-02-16

**Authors:** Eugene Strativnov

**Affiliations:** Gas Institute of NAS of Ukraine, 39, Degtyarivska str, Kiev, 03113 Ukraine

**Keywords:** Activation, Activated coal, Absorbent, Ecology, Cleanup, Pollution, Simulation, ANSYS, SolidWorks

## Abstract

We propose the technology of obtaining the promising material with wide specter of application-activated nanostructured carbon. In terms of technical indicators, it will stand next to the materials produced by complex regulations with the use of costly chemical operations. It can be used for the following needs: as a sorbent for hemosorption and enterosorption, for creation of the newest source of electric current (lithium and zinc air batteries, supercapacitors), and for processes of short-cycle adsorption gas separation.

In this study, the author gives recommendations concerning the design of the apparatus with fluidized bed and examples of calculation of specific devices. The whole given information can be used as guidelines for the design of energy effective aggregates. Calculation and design of the reactor were carried out using modern software complexes (ANSYS and SolidWorks).

**Electronic supplementary material:**

The online version of this article (doi:10.1186/s11671-017-1843-0) contains supplementary material, which is available to authorized users.

## Background

Activated carbon is a porous material produced from different carbonaceous materials: charcoal, coal and petroleum coke, coconut shell, etc. [1]. The essence of activation consists in the pores’ opening that is in the closed state in the carbon material. It is executed by a thermo-chemical method (the material is impregnated with a solution of zinc chloride, potassium carbonate, or some other compounds and is heated without access of air) or by treatment with superheated steam or carbon dioxide at a temperature of 800–850 °C. The most widespread activation method is simultaneous supply of incomplete combustion of natural gas and steam in certain proportions into the activation apparatus. The specific surface of the pores of the activated carbon is the most important indicator of its quality, which can reach 600 to 2200 m^2^ per gram, depending on the initial material and activation methods. Thanks to its absorption properties, activated carbon is one of the most common materials which are widely used in various industries: ecology, medicine, civil defense, and military sphere (for example, in the manufacturing of respirators and life-support systems of air-RAID shelters). Recently, the manufacture of rechargeable batteries of a “new generation” [2] is very important in the production of which activated carbon with high purity and specific surface area is also used.

### The Reactor Type and Its Key Elements: Hydrodynamic Characteristics of Particles of the Processed Material [3, 4]

**Fig. 1:**
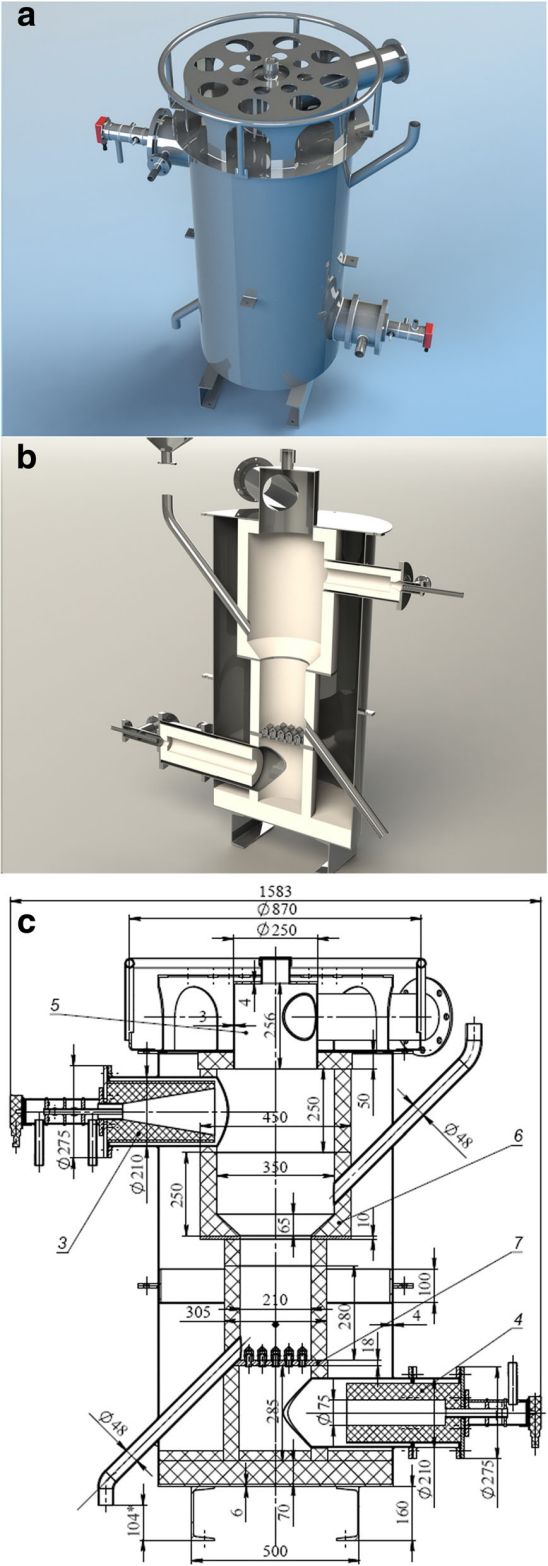
High temperature coal activation reactor. **a** External view of the reactor. **b** Visualization of the cross section of the reactor. **c** Draft of the cross section of the reactor

If the quality and uniformity of the final product are the most important factors, it is necessary to apply the apparatus of periodic action. However, this type of apparatus has some drawbacks, for example, the complexity of technological modes of operation and the constructional complexity of loading and unloading elements of the unit. Gas distribution grid of a “cap” type is used to maximize the efficiency of the reactor of fluidized bed. The main function of the gas distribution grid is uniformly supplying of fluidized gases and generating of the corresponding hydrodynamic mode (depending on the design, this element can be manufactured by a porous material or by caps with holes). This key element of the unit is exposed to extreme temperatures. This in turn places high demands to the used materials of which it is made. The internal volume of activation reactor consists of three main zones: the area under the gas distribution grid, the area above the gas distribution grid (zone of fluidization), and the separation space. The products of incomplete combustion of natural gas are mixed with water vapor in a specific ratio in the first zone of reactor’s volume. In the second zone, gas velocity must guarantee initial material fluidizing (initial material at the beginning of the activation process is more dense and larger than at the end of the process). In the third zone, the cross section of the reactor is increased to reduce gas speed which passes through the zone. The gas speed in this zone should provide the return of the main part of the small and light materials in to the second zone by gravitational forces. The external view of the reactor of activation and its cross section are shown in Fig. 1a–c.

**Fig. 2:**
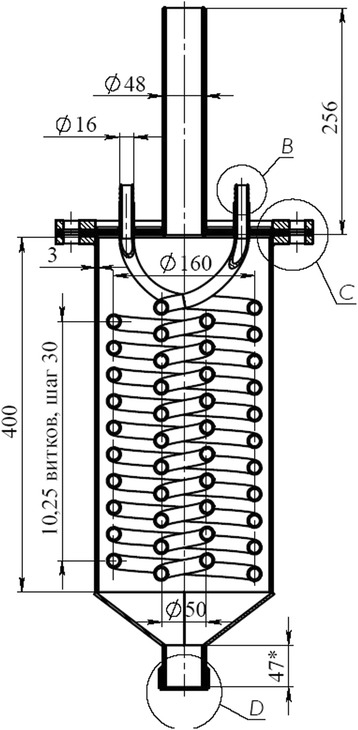
Hermetic cooler for activated coal

At the process of coal activation, combustible gases are emitted which are burnt in the separation zone. For this purpose, an additional burner or air supply is used. This element is not principle, and it will not be considered in this article. After material processing, the activated carbon has to be unloaded from the reaction zone and must be cooled without oxygen access. For this purpose, the hermetic cooler has been applied (Fig. 2).

Usually, the source material is crushed and sieved. Different types of coal have different densities and particle size distribution before and after the activation process. For example, the size of the particles prior to activation may be 0.25 to 10 mm and after the process is less than 0.1 mm. The density of the activated carbon of various types is in the range from 300 to 500 g per liter. In this regard, the design and calculation of appropriate reactor zones are necessary to consider as indicators together with the required performance of the unit.

## Methods

### Calculation Methodology of Material Flows and the Main Parameters of the Unit

The calculation of the material flows and basic dimensions of the reactor (and vice versa) has a certain logic and consistency. Based on the performance of the unit, you must specify the diameter of the reaction zone (diameter of the gas distribution grid). Similarly, it is necessary to determine the “flying speed” of the material based on its type. The example of calculation of activation reactor is given below.

#### Example of Calculating Flows

- The rate of fluidization of the feedstock with the particulate composition 1…3 mm in the reaction zone *W*_f_ = 0.5 m/s (the speed of free fall *W*_f_ = 1 m/s)
- The area of the reaction zone for the apparatus with a capacity of 3 cm^3^/h *S* = 0.785 × *D*2 = 0.785 × of 0.212 = 0.0346 m^2^
- Rate of flue gases (60%) and steam (40%) *G* = *S* × *W* = 0.0346 × 0.5 = 0.0173 m^3^/s (62.28 m^3^)
- Steam consumption of *G*_steam_ = *G* × 0.4 = 0.00692 m^3^/s (24.9 m^3^/h) or 14.9 l/h (1 kg water = 1.67 m^3^ of steam)
- Flow flue gas *G*_f.gas_ = G × 0.6 = 0.01038 m^3^/s (37.368 m^3^/h)
- Coefficient of thermal expansion *K* = (273 + *t*)/293 = (273 + 900)/293 = 4
- The consumption of the combustible mixture *G*_comb.mix_ = *G*_comb.mix_/4 = 0.002595 m^3^/s

Hence, the calculated consumption of fuel and air is:

- Gas consumption *G*_gas_ = *G*_comb.mix_ × 0.1 = 0.0002595 m^3^/s (0.9342 m^3^/h)
- Air flow *G*_air_ = *G*_comb.mix_ × 0.9 = 0.0023355 m^3^/s (8.41 m^3^/h)

**Fig. 3:**
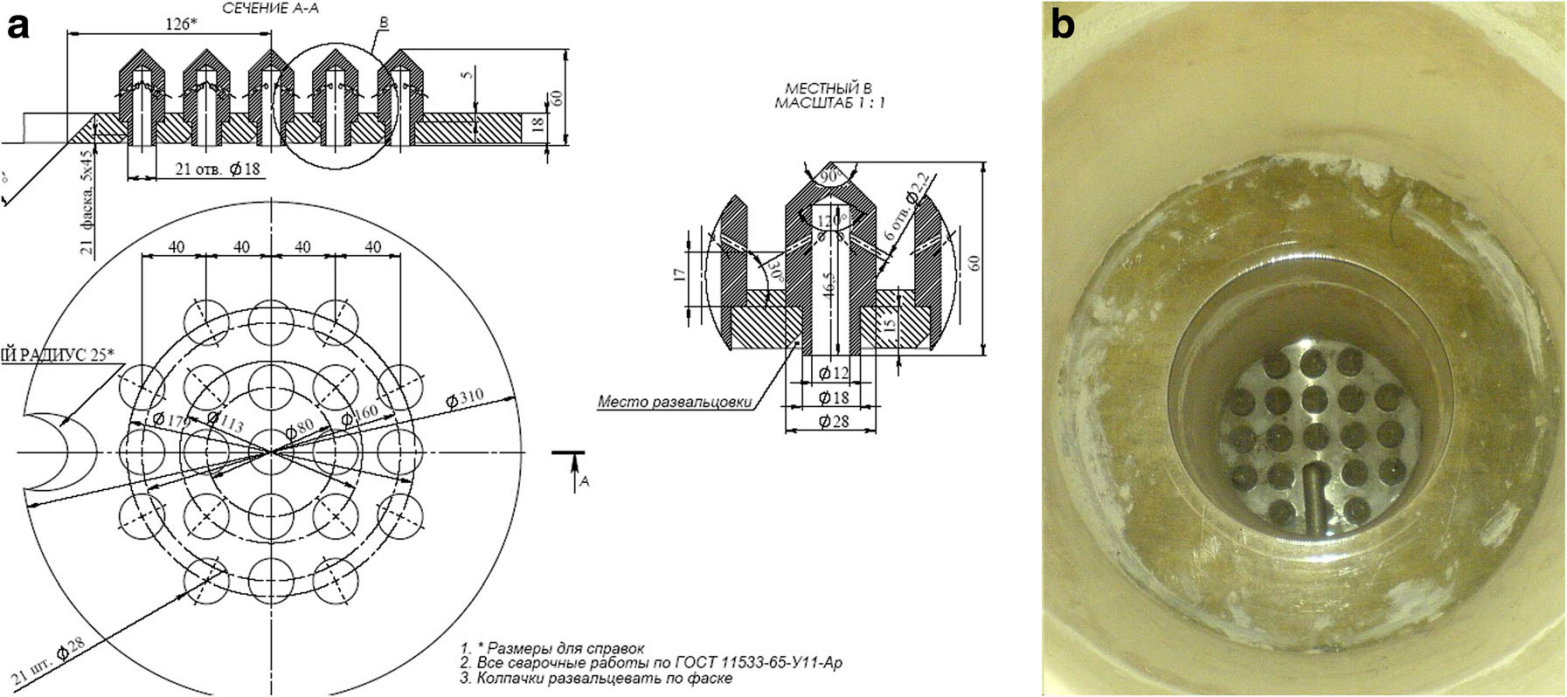
Gas distribution grid. **a** Drawing of the gas distribution grid. **b** External view of the gas distribution grid

For more reliable operation, we apply the gas distribution grid which consists of special caps. The design of the gas distribution grid and appearance are shown in Fig. 3.

The number of holes in the cap is six pieces to ensure the material fluidizing the “living section” of the grid should be 1–3% of the grid area (reaction zone). The calculation of the gas distribution grid is concluded to determine the number of caps and the diameter of the holes in them.

#### Example of Calculating Hydrodynamic

- Square of grid (reaction zone) *S* = 0.785 × *D*2 = 0.785 × of 0.212 = 0.0346 m^2^
- 2%, it is 6.92 × 10–4 m^2^ (21 caps)
- The cross-sectional area of one cap is 3.29 mm^2^ (six holes)
- The area of one hole is 5.5 × 10-6 m
- The diameter of the hole of the cap is *D* = (5.5 × 10–6/0.785)0.5 = 2.64 mm, standard diameter of holes of 2.2 mm
- Thus:
- The gas flow rate of a single hole cap is *G* = 0.0173/(21 × 6) = 1.37 × 10–4 m^3^/s
- The cross-sectional area of one hole of the cap is *S* = 0.785 × 2.22 = 3.8 × 10–6 m^2^
- The calculated average exit speed from the holes of fluidizing agent (flue gas + steam) is *W* = *G*/*S* = 36 m/s (the recommended starting speed is 35…40 m/s).

**Fig. 4:**
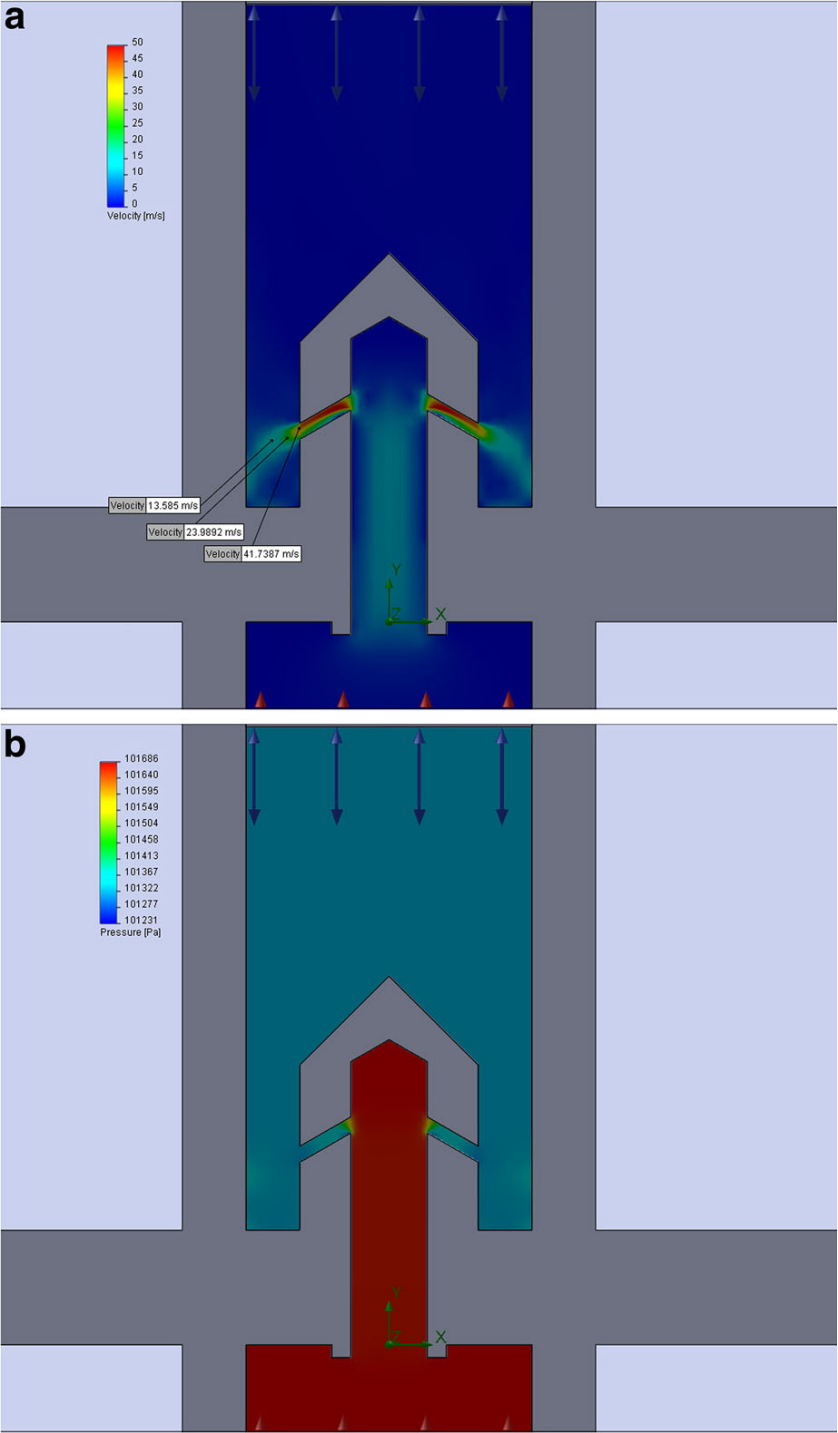
Simulation of gas flow in the holes of the cap of the gas distribution grid. **a** The velocity of gas flow. **b** Hydraulic resistance of one cap of gas distribution grid

With the help of computer simulation [5, 6], test calculations of the velocities encountered in the hole of the cap are performed (see Fig. 4a). Calculation of hydraulic resistance arising from the passage of hot gases through the holes in the cap is shown in Fig. 4b.

### CFD Simulation of Steam-Gas Mixture Flows in the Key Zones of the Reactor Activation

**Fig. 5:**
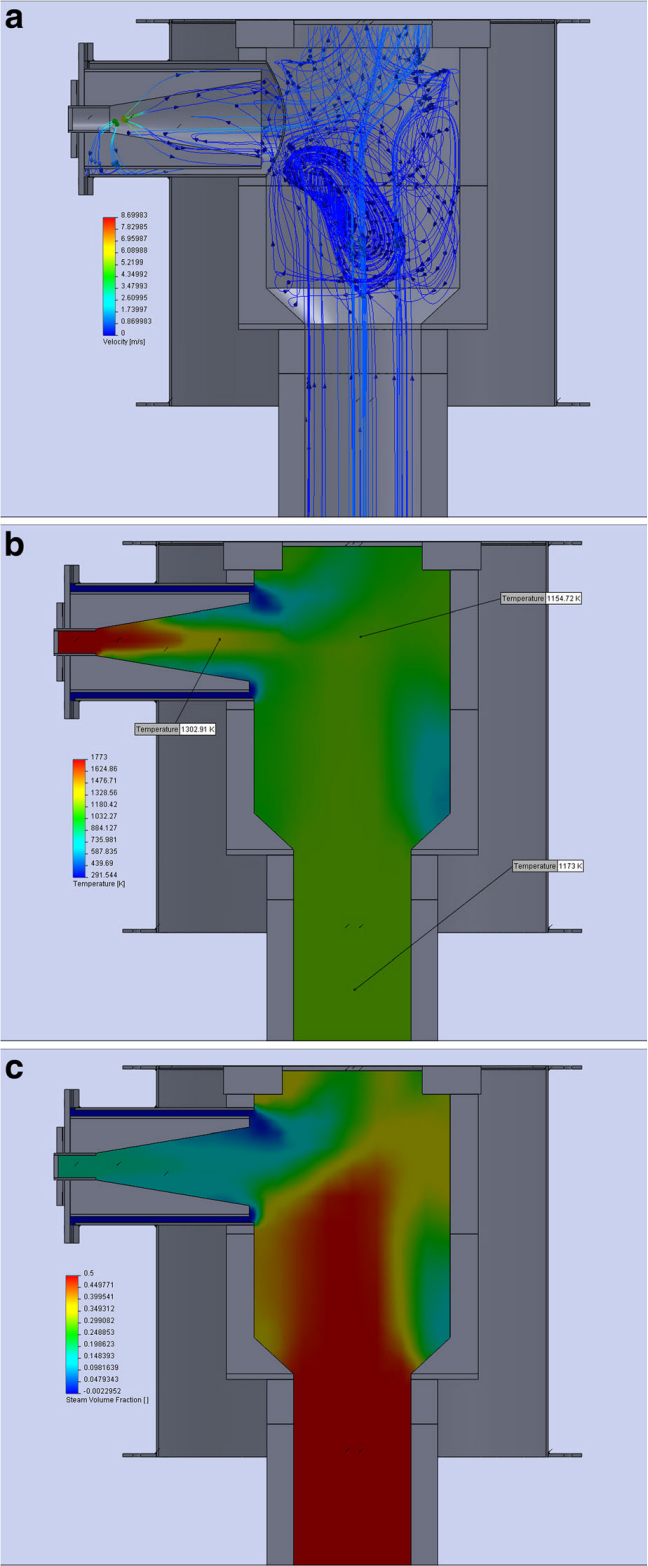
Simulation of gas flow in separating zone of activation reactor. **a** Velocity and direction of flows. **b** Distribution of temperature. **c** Distribution of steam concentration

Sustainable fluidization (at the beginning of the activation process) and minimizing of particles removal (at the end of the process) in the appropriate zones of the activation reactor, in the area of fluidizing zone and in the separation zone, are achieved due to calculations and simulations of the processes in these zones [5, 6]. Having data about the volume of material flows in the reactor, it is rational to simulate their motion in the space of the designing unit to identify “dead” zones and flow turbulence. Figure 5a–c shows the velocity and direction of flows, plot of the temperature distribution, and the concentration of water vapor in the separation zone.

**Fig. 6:**
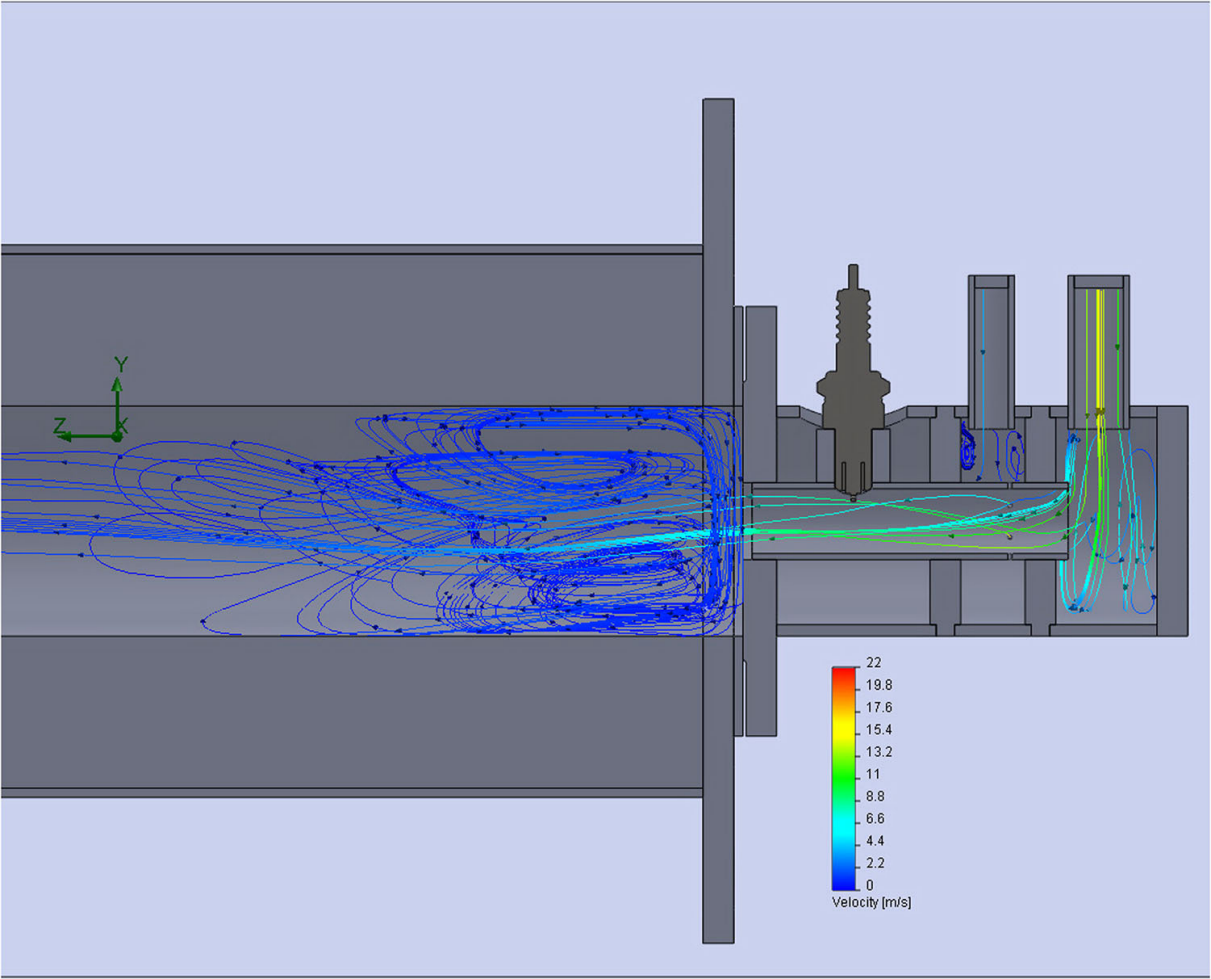
Simulation of flow velocity in the burner

Burner device is used for generating heat by gas fuel burning. The fuel efficiency is achieved, thanks to a good mixing of natural gas and oxidant. The sudden expansion of the channel serves the ignition and stabilization of combustion in the combustion chamber. To make sure of the “quality” vortices, it is possible by simulation [5, 6] of cold gas flows (in case of burning the volume of gases and respectively turbulence are guarantee increased) as shown in Fig. 6.

**Fig. 7:**
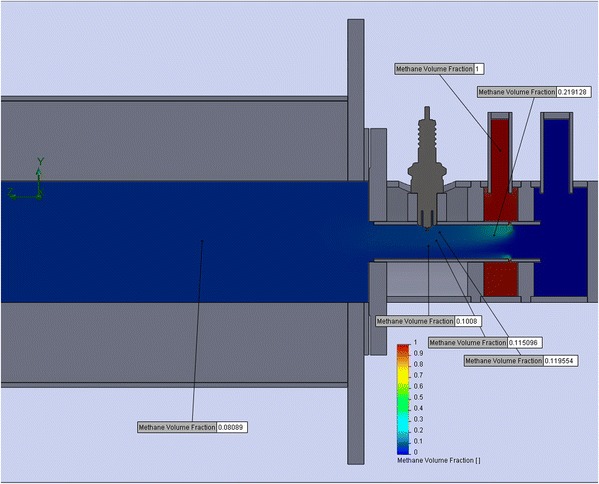
Simulation of methane concentration in the burner

Modeling the mixing process of air and fuel allows you to accurately determine a specific area in the mixing chamber. The concentration of the components will be flammable in this area. Accordingly, in this precise area, it is advisable to place the ignition device of the burner (see Fig. 7).

### CFD Simulation of Heat-Gas Mixing of Gas-Vapor Mixture in the Area under the Gas Distribution Grid in order to Align the Temperature Field on Its Surface

**Fig. 8:**
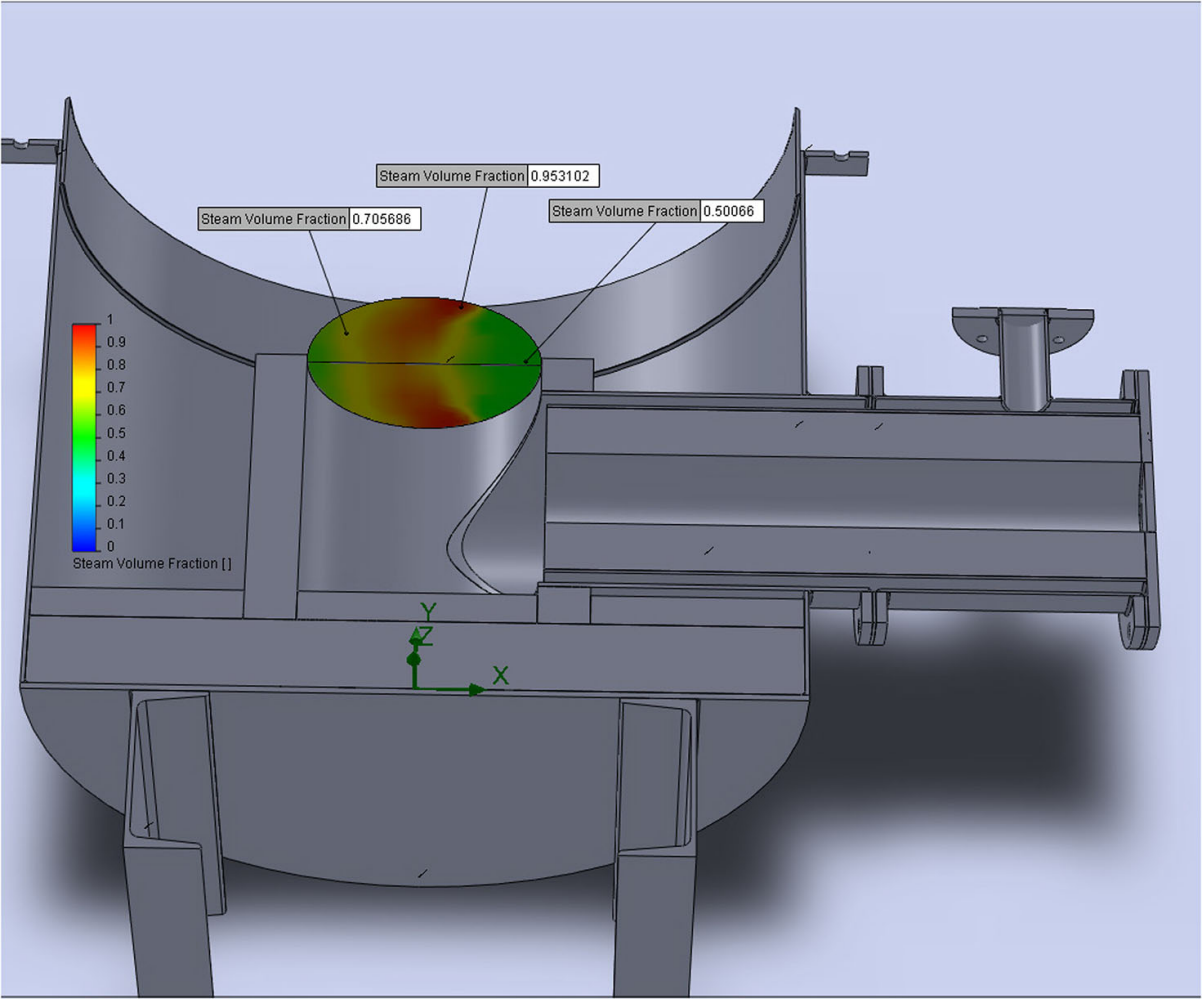
Temperature distributions at the bottom surface of the gas distribution grid (variant one of the burner stone construction)

As mentioned above, the gas distribution grid of the reactor is the most heat-stressed and critical detail of this device. At the process, it is exposed to high temperatures (violation of the mode rules, the temperature of the gas distribution grid can reach 1500 °C) and sharp gradient of temperature on the surface of the grid. In addition, products of a natural gas burner and steam acts on it chemically. Hot gases and the cold vapor gases passing through the gas distribution grid in order to avoid its destruction must be thoroughly mixed. Figure 8 shows the degree of mixing of gases on the bottom surface of the gas distribution grid.

**Fig. 9:**
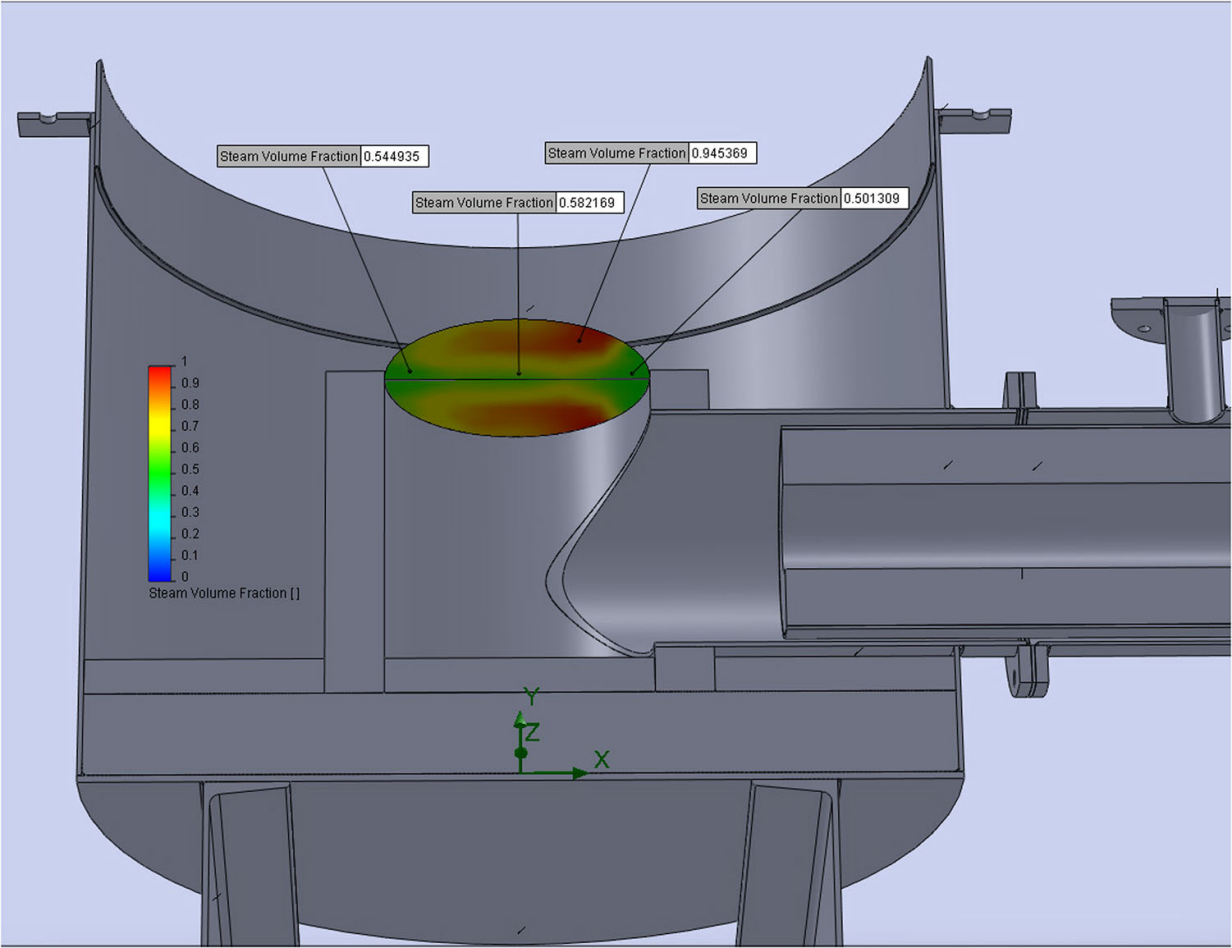
Simulation of temperature distribution at the bottom of gas distribution grid (variant two of the burner stone construction)

This simulation showed that at the bottom surface of gas distribution grid, there is a great temperature gradient. Thanks to the above information, it was decided to change the design of the burner stone (to reduce its length) for better mixing of steam and combustion products. As a result of the mentioned design changes on the bottom surface of the gas distribution grid, we managed to achieve less temperature gradient due to better mixing of the flows (see Fig. 9).

**Fig. 10:**
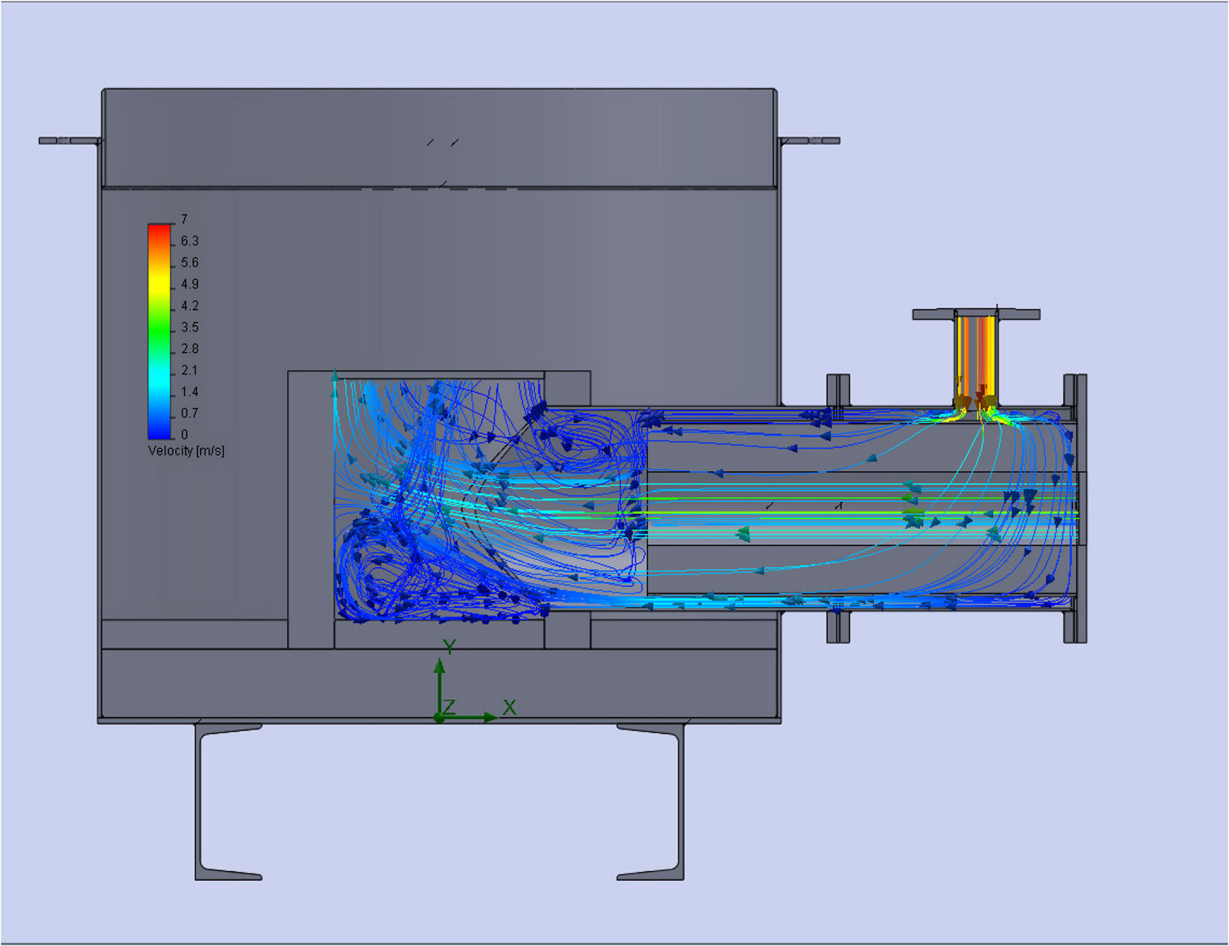
Direction and velocity simulation of combustion products flows and steam in the mixing chamber

The direction and velocity of the flow in the mixing chamber of vapor and combustion gases are shown in Fig. 10.

3-D designing of the reactor and its individual elements combined with the modeling of physical processes occurring in the reactor manage to avoid the conceptual errors at the initial stage of the manufacturing of the device. Such errors would result to rapid failure of the whole unit and therefore to great financial losses for the enterprise.

### Design of the Unit in 3-D (SolidWorks); Strength Calculation of Construction Elements of Activation Unit (ANSYS)

**Fig. 11:**
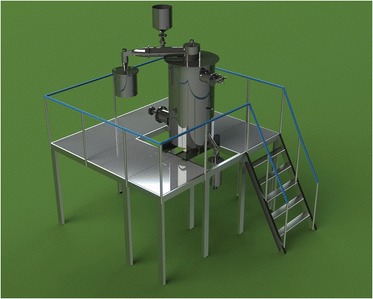
Visualization of frame for services of activation reactor

Knowing the mass of the reactor, it is possible to design a carrier frame and a platform for maintenance of unit’s elements and auxiliary equipment (Fig. 11).

**Fig. 12:**
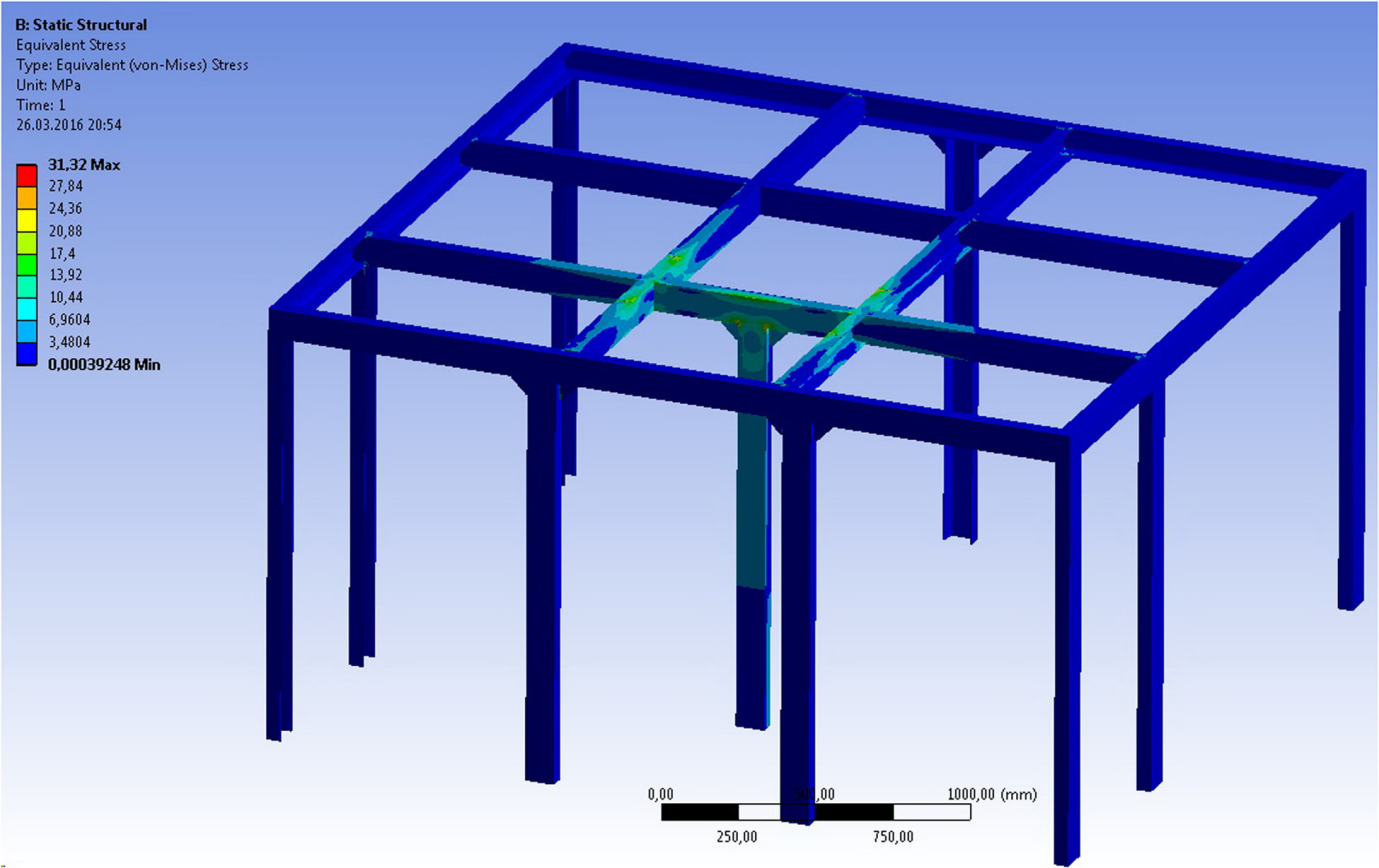
Checking calculation of strength of the supporting frame for equipment maintenance

The calculation test [6] of the supporting frame on a strength showed a peak stress in the material of 31.3 MPa, which gives the opportunity to use cheaper steel or to reduce metal consumption of the design (the yield strength for cheap carbon steel is 220 MPa) (see Fig. 12).

### Running and Commissioning of the Activation Reactor for Different Types of Processing Materials

**Fig. 13:**
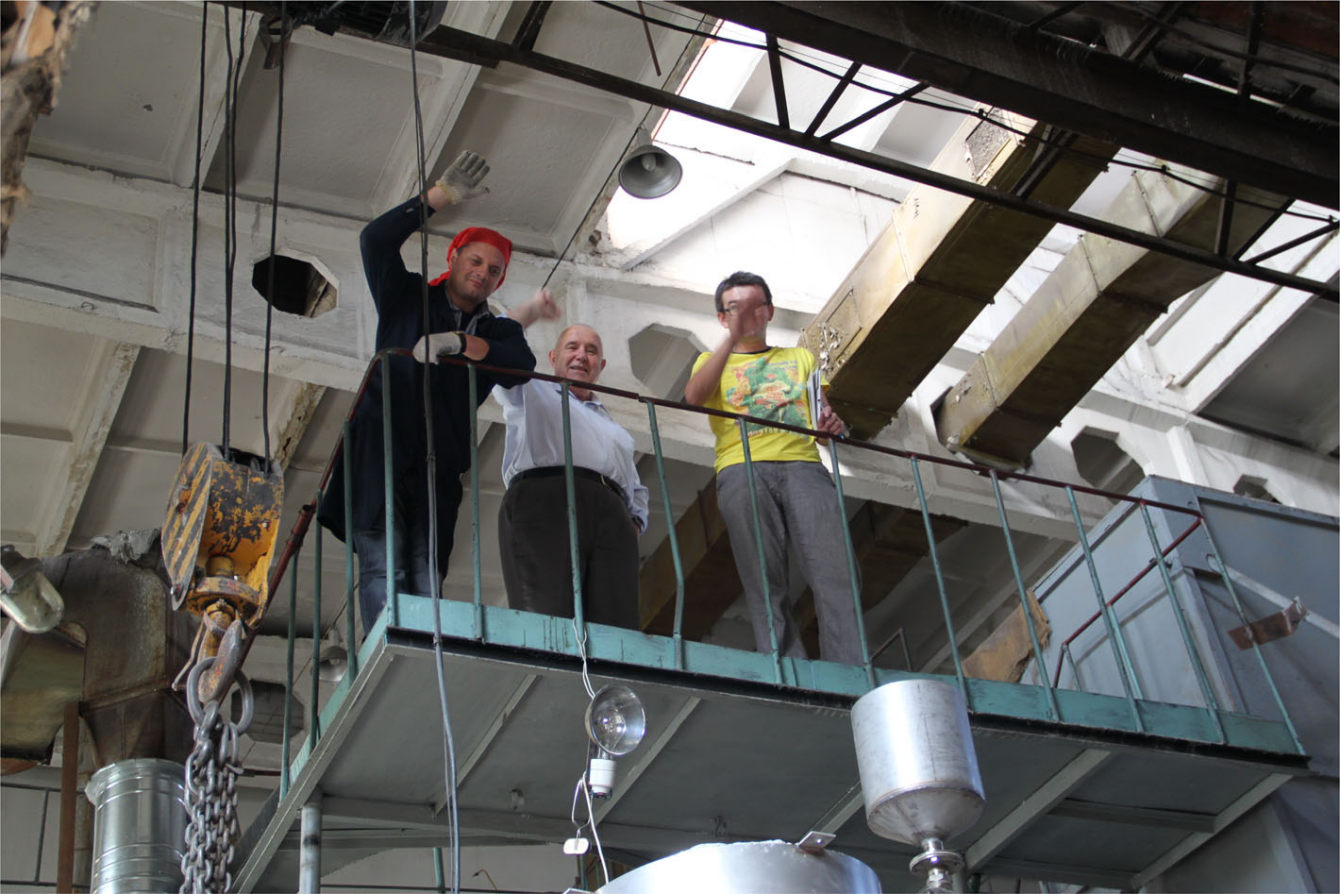
Successful running of the unit (from *left* to *right*: the author, Eugene Strativnov, Director of the Institute of gas of NASU, Borys Bondarenko, and colleague of author, Alexander Khovavko)

Preparing of the unit to work is concluded in its connection to the mains, water supply, and air and gas lines, as well as verification of the steam generator and other accessory before you turn them on. Next, the reactor will start and run to a nominal mode of air, gas, and steam consumption. Then, the material is supplying the activation. Figure 13 shows a solemn moment of demonstration of unit operation (from left to right: the author, Eugene Strativnov, Director of the Gas Institute of NASU, Borys Bondarenko, and colleague, Alexander Khovavko).

**Fig. 14:**
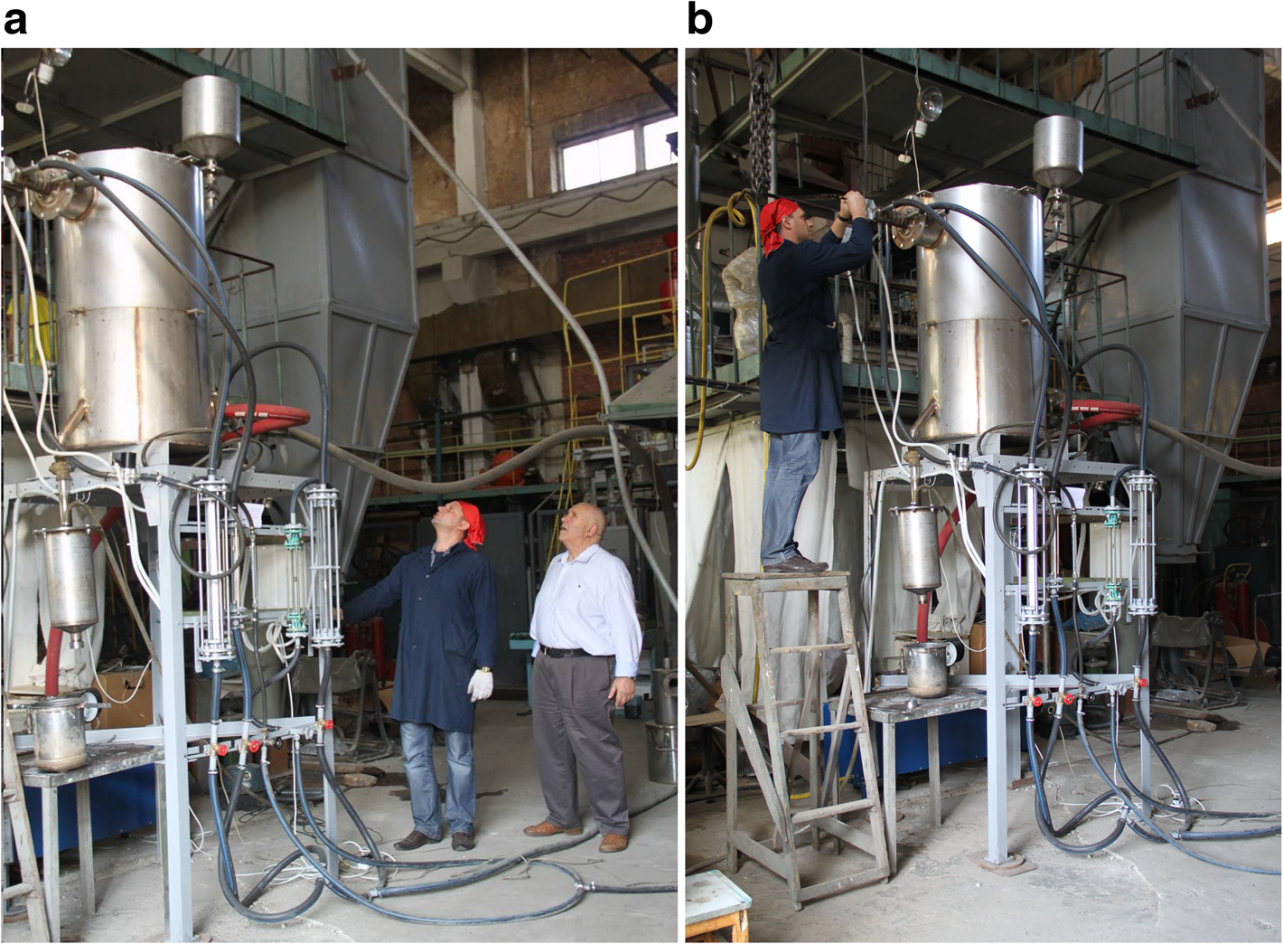
Set-up and tuning of equipment. **a** Set-up of gas and air consumption. **b** Tuning of burner automatic system

Figure 14 shows the stages of commissioning of the unit.

## Results and discussion

**Fig. 15:**
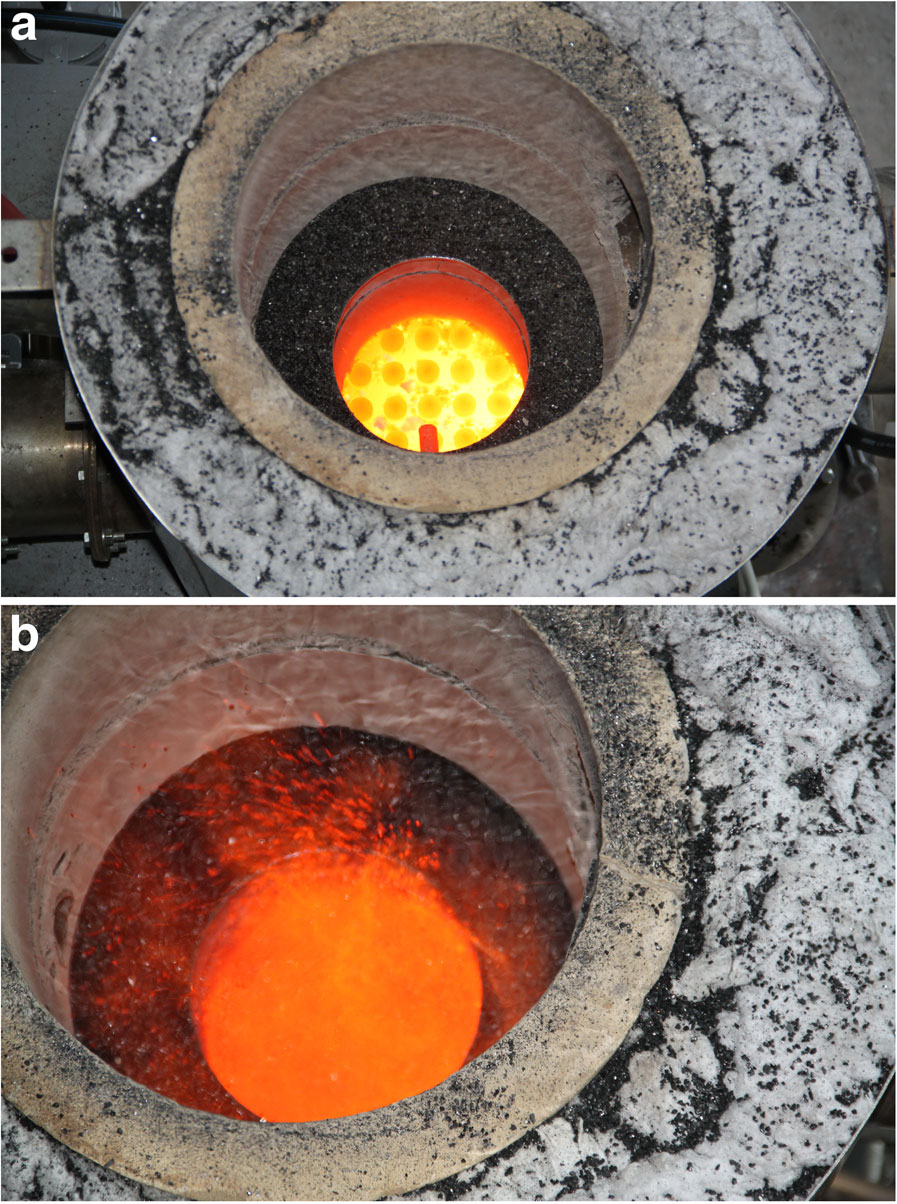
Gas distribution grid in the processes. **a** Hot distribution grid. **b** Fluidized bed of activated carbon



The first runs of the reactor were made without the protective cover. Thanks to this, we managed to fix by photo and video the heated gas distribution grid without coal and directly the activation process (see Fig. 15a, b) (Additional file 1: Video S1).

**Additional file 1: Video S1.** This video shows the operation of the reactor and stable boiling of the material in it. (mov 157483 kb)


### Technical Characteristics of the Activation Unit

- Productivity on the finished product—1..0.3 kg/h (depending on the type of raw materials;
- The natural gas consumption is 1 m^3^/h
- Steam consumption is 10…15 l/h
- The working temperature of the activation process is 900 °C
- The density of activated carbon is 0.42…0.5 g/sm^2^
- The specific surface of the coal is 1300…2000 m^2^/g (depending on the raw material and activation mode)
- The device is of periodic action (duration of one cycle 1…2 h)

## Conclusions

The Gas Institute of NAS of Ukraine is a leading scientific institution that has many years of experience in the development and implementation of technologies and technological equipment for the production of activated carbon on an industrial level. Modern and energy-saving units of the new generation are the result of many years of experience and CFD technologies. For example, described in this article modern unit which implementation is carried out in the USA (Argonne National Laboratory) in 2013. The Gas Institute of NAS of Ukraine is constantly working on even more sophisticated, resource-saving technologies and equipment to produce high-quality activated carbon from different raw materials.

## Abbreviations

CFD: Computational fluid dynamic
